# Reprogramming of Small Noncoding RNA Populations in Peripheral Blood Reveals Host Biomarkers for Latent and Active Mycobacterium tuberculosis Infection

**DOI:** 10.1128/mBio.01037-19

**Published:** 2019-12-03

**Authors:** Leonardo Silva de Araujo, Marcelo Ribeiro-Alves, Thyago Leal-Calvo, Janaína Leung, Verónica Durán, Mohamed Samir, Steven Talbot, Aravind Tallam, Fernanda Carvalho de Queiroz Mello, Robert Geffers, Maria Helena Féres Saad, Frank Pessler

**Affiliations:** a Research Group Biomarkers for Infectious Diseases, TWINCORE Centre for Experimental and Clinical Infection Research, Hannover, Germany; b Fundação Oswaldo Cruz (Fiocruz), Rio de Janeiro, Brazil; c Helmholtz Centre for Infection Research, Braunschweig, Germany; d Thoracic Diseases Institute, Federal University of Rio de Janeiro, Rio de Janeiro, Brazil; e Department of Zoonoses, Faculty of Veterinary Medicine, Zagazig University, Zagazig, Egypt; f Institute for Laboratory Animal Science, Hannover Medical School, Hannover, Germany; g Institute for Experimental Infection Research, TWINCORE Centre for Experimental and Clinical Infection Research, Hannover, Germany; h Centre for Individualised Infection Medicine, Hannover, Germany; INTA; Washington University School of Medicine in St. Louis

**Keywords:** biomarkers, biosignature, miRNA, incipient tuberculosis, piRNA, RNA, snRNA, sncRNA, snoRNA, subclinical tuberculosis, transcriptome, tuberculosis

## Abstract

In tuberculosis (TB), as in other infectious diseases, studies of small noncoding RNAs (sncRNA) in peripheral blood have focused on microRNAs (miRNAs) but have neglected the other major sncRNA classes in spite of their potential functions in host gene regulation. Using RNA sequencing of whole blood, we have therefore determined expression of miRNA, PIWI-interacting RNA (piRNA), small nucleolar RNA (snoRNA), and small nuclear RNA (snRNA) in patients with TB (*n* = 8), latent TB infection (LTBI; *n* = 21), and treated LTBI (LTBItt; *n* = 6) and in uninfected exposed controls (ExC; *n* = 14). As expected, sncRNA reprogramming was greater in TB than in LTBI, with the greatest changes seen in miRNA populations. However, substantial dynamics were also evident in piRNA and snoRNA populations. One miRNA and 2 piRNAs were identified as moderately accurate (area under the curve [AUC] = 0.70 to 0.74) biomarkers for LTBI, as were 1 miRNA, 1 piRNA, and 2 snoRNAs (AUC = 0.79 to 0.91) for accomplished LTBI treatment. Logistic regression identified the combination of 4 sncRNA (let-7a-5p, miR-589-5p, miR-196b-5p, and SNORD104) as a highly sensitive (100%) classifier to discriminate TB from all non-TB groups. Notably, it reclassified 8 presumed LTBI cases as TB cases, 5 of which turned out to have features of Mycobacterium tuberculosis infection on chest radiographs. SNORD104 expression decreased during M. tuberculosis infection of primary human peripheral blood mononuclear cells (PBMC) and M2-like (*P* = 0.03) but not M1-like (*P* = 0.31) macrophages, suggesting that its downregulation in peripheral blood in TB is biologically relevant. Taken together, the results demonstrate that snoRNA and piRNA should be considered in addition to miRNA as biomarkers and pathogenesis factors in the various stages of TB.

## Abstract (precis)

Tuberculosis is the infectious disease with the worldwide largest disease burden and there remains a great need for better diagnostic biomarkers to detect latent and active M. tuberculosis infection. RNA molecules hold great promise in this regard, as their levels of expression may differ considerably between infected and uninfected subjects. We have measured expression changes in the four major classes of small noncoding RNAs in blood samples from patients with different stages of TB infection. We found that, in addition to miRNAs (which are known to be highly regulated in blood cells from TB patients), expression of piRNA and snoRNA is greatly altered in both latent and active TB, yielding promising biomarkers. Even though the functions of many sncRNA other than miRNA are still poorly understood, our results strongly suggest that at least piRNA and snoRNA populations may represent hitherto underappreciated players in the different stages of TB infection.

## INTRODUCTION

Tuberculosis (TB) has affected humankind for more than 4,000 years and persists to this day as a leading cause of death (1, 2). Mycobacterium tuberculosis, the causative agent of TB, has adapted to survive in the human host, causing latent TB infection (LTBI) that is maintained for long periods of time (3, 4). The outcome of LTBI ranges from complete clearance of infection to stable latency and low-grade TB symptoms all the way to disseminated disease (5). Although there are well-established methods to diagnose TB, they have well-known drawbacks such as insufficient sensitivity (sputum smear), long (4-to-8-week) turnaround time to results (culture) (3), and inability to differentiate LTBI from TB (tuberculin skin test [TST], interferon gamma [IFN-γ] release assay [IGRA]) (6, 7). Even the recently developed Xpert M. tuberculosis/RIF assay has been reported to exhibit limited sensitivity in detection of early progressors or paucibacillary TB cases (8). Therefore, a better understanding of the core cellular/molecular mechanisms responsible for the pathogenesis of active TB and of the progression of LTBI to active disease would contribute to improved recognition of individuals at high risk of progression before a clinical diagnosis of TB is made and would lead to novel interventions to ameliorate the consequences of active disease.

MicroRNAs (miRNAs) constitute key regulators of gene expression at the posttranscriptional level; they have well-known effects on pathogenesis and immune responses in infectious diseases and offer potential uses as diagnostics (9, 10). Due to their small size (∼22 nucleotides [nt]) and molecular structure, they are also expected to be more-stable biomarkers than mRNA (9, 11). Indeed, several recent reports have suggested that changes in host miRNA expression represent a hallmark of bacterial infections in humans and animals at both the cellular and organismal levels (12), including in M. tuberculosis infection (13–15).

Other major classes of small noncoding RNAs (sncRNA) also contribute to the regulation of gene expression (16), i.e., PIWI-interacting RNAs (piRNA), small nucleolar RNAs (snoRNA), and small nuclear RNAs (snRNA). The principal characteristic of piRNA is their capability to bind to PIWI proteins. Their functions were initially believed to be restricted to antiviral defenses in insects and to maintaining genome stability in germ line cells; however, it now appears that piRNA expression is also highly regulated in somatic cells in response to a variety of stimuli (16, 17). For instance, previous studies revealed that piRNA are abundant in human CD4^+^ primary T lymphocytes and that piR_30840 was able to bind to pre-mRNA introns via sequence complementarity, resulting in a significant downregulation of interleukin-4 production (18). snoRNA are 60 to 300 nt in length and play important roles in post-transcriptional modifications during ribosomal assembly. They can be classified into two families according to the presence of a conserved H/ACA box (SNORA) or C/D box (SNORD) sequence (17, 19, 20), and dysregulation in their expression can be part of stress responses and disease mechanisms (19, 21). A recent report showed increased SNORD61 levels in plasma from TB patients after an effective 24-week anti-TB treatment, providing the first evidence of a potential role in host responses in TB (22). snoRNA precursors can give rise to miRNA and piRNAs, thus adding another important biological function to this class of sncRNA (23). snRNA (∼150 nt) are part of small ribonucleoprotein particles and are important for the positioning of the spliceosome on the substrate pre-mRNA (17). Some snRNA have relatively stable expression, as exemplified by the uridine-rich snRNA RNU6, which is often used as a constitutively expressed internal control for quantitative PCR (qPCR) amplification of small RNAs (24).

Despite the potential importance of all major sncRNA classes to the pathogenesis of TB, work has thus far focused on miRNAs; there are no published studies on any sncRNA as biomarkers to identify LTBI among recently exposed contacts (rCt) and no TB studies featuring simultaneous profiling and computational evaluation of a broader set of sncRNA classes. Besides aiming to identify new RNA biomarkers for TB, we therefore also conducted this study to improve our understanding of the differences in the relative degrees of reprogramming of the various sncRNA populations in a major human infectious disease. To this end, we used integrative small RNA sequencing (RNAseq) to characterize differences in the expression levels of miRNA, piRNA, snoRNA, and snRNA in whole-blood samples from individuals with LTBI and active TB in comparison with exposed controls (ExC) and treated LTBI (LTBItt) subjects.

## RESULTS

### Characteristics of the study cohort.

Selected demographic and clinical data of the study participants are summarized in Table 1 (for a detailed description, see Table S1 in the supplemental material). The diagnostic groups did not differ in terms of age or sex. Responses to the TST and short-term IGRA (st-IGRA) and long-term IGRA (lt-IGRA) screening tests differed greatly within both the LTBI and LTBItt groups but did not differ between these two groups. Nonspecific chest X-ray (CXR) changes were observed in 4 participants in the LTBI and LTBItt groups combined, whereas abnormalities consistent with M. tuberculosis infection were observed in 7 of the 8 TB patients and spanned a range of lung injury. The one patient without such CXR findings had pleural TB. CXR abnormalities suggestive of TB were also reported in 5/21 LTBI cases, but results of bacterial investigations were negative (see Table 3; see also Table S1). Half of the TB patients were sputum smear negative for AFB, but all TB cases had a positive sputum culture, except for the single patient with pleural TB, who was diagnosed by pleural tissue culture. Six of the eight index cases reported respiratory symptoms consistent with TB. Similar symptoms were present in the other biological groups, although less frequently.

**TABLE 1:** Characteristics of the study participants^*a*^

Supergroup or study group	*n*	No. (%) of males	Mean (SD) values^*b*^				TST/IGRA response	No. (%) of patients with indicated CXR abnormality			No. (%) of patients with indicated response consistent with TB		
			Age (yrs)	TST (mm)^*f*^	st-IGRA (pg/ml)	lt-IGRA (pg/ml)		Nonspecific	Probability of M. tuberculosis infection^*c*^		Respiratory symptom(s)	AFB^+^	Culture^+^
									Possible	Likely			
NTB													
ExC	14	5 (35.7)	35.6 (13)	0.6 (1.4)	0.1 (0.4)	6.8 (10.7)	−/−	0	0	0	4 (28.6)	NA	NA
LTBI	21	7 (33.3)	46.1 (10.5)	14.3 (5.0)***	25 (57.9)**	561.2 (584.1)***	+/+	3 (14.3)	2 (9.5)	3 (14.3)	9 (42.9)	0	0
LTBItt^*d*^	6	3 (50)	38.5 (10.8)	9.5 (2.9)^#^	31.2 (42.6)*	368.3 (347.60)**	+/+	1 (16.7)	0	0	2 (33.3)	0	0
TB	8	5 (62.5)	47 (18.2)	8.7 (4.3)^#^	NA	NA	NA	0	1 (12.5)	6 (75)	6 (75)	4 (50)	8 (100)^*e*^

Total	49	20 (40.8)	42.3 (13.7)	—	—	—	—	4	3	9	21	4	8

a Abbreviations: ExC, exposed controls; LTBI, latent TB infection; LTBItt, treated LTBI; TB, tuberculosis; NTB, non-TB; NA, not available; —, not done; TST, tuberculin skin test; st-IGRA, short-term IGRA; lt-IGRA, long-term IGRA; +, positive; −, negative.

b Statistical significance of results of multiple-group comparisons is indicated as follows: ^#^, *P* ≤ 0.08; *, *P* < 0.05; **, *P* < 0.005; ***, *P* < 0.001 (versus ExC).

c Infiltrates (with or without cavitation) or fibrotic scars consistent with TB.

d Screening tests for LTBI/TB were performed before IPT and were not repeated.

e One case was detected by positive culture of the pleural tissue sample.

f Induration diameter.

10.1128/mBio.01037-19.6 TABLE S1 Detailed characteristics of the study population. Download Table S1, XLSX file, 0.02 MB. Copyright © 2019 de Araujo et al. 2019 de Araujo et al. This content is distributed under the terms of the Creative Commons Attribution 4.0 International license.

### RNAseq: counting statistics.

**FIG 1:**
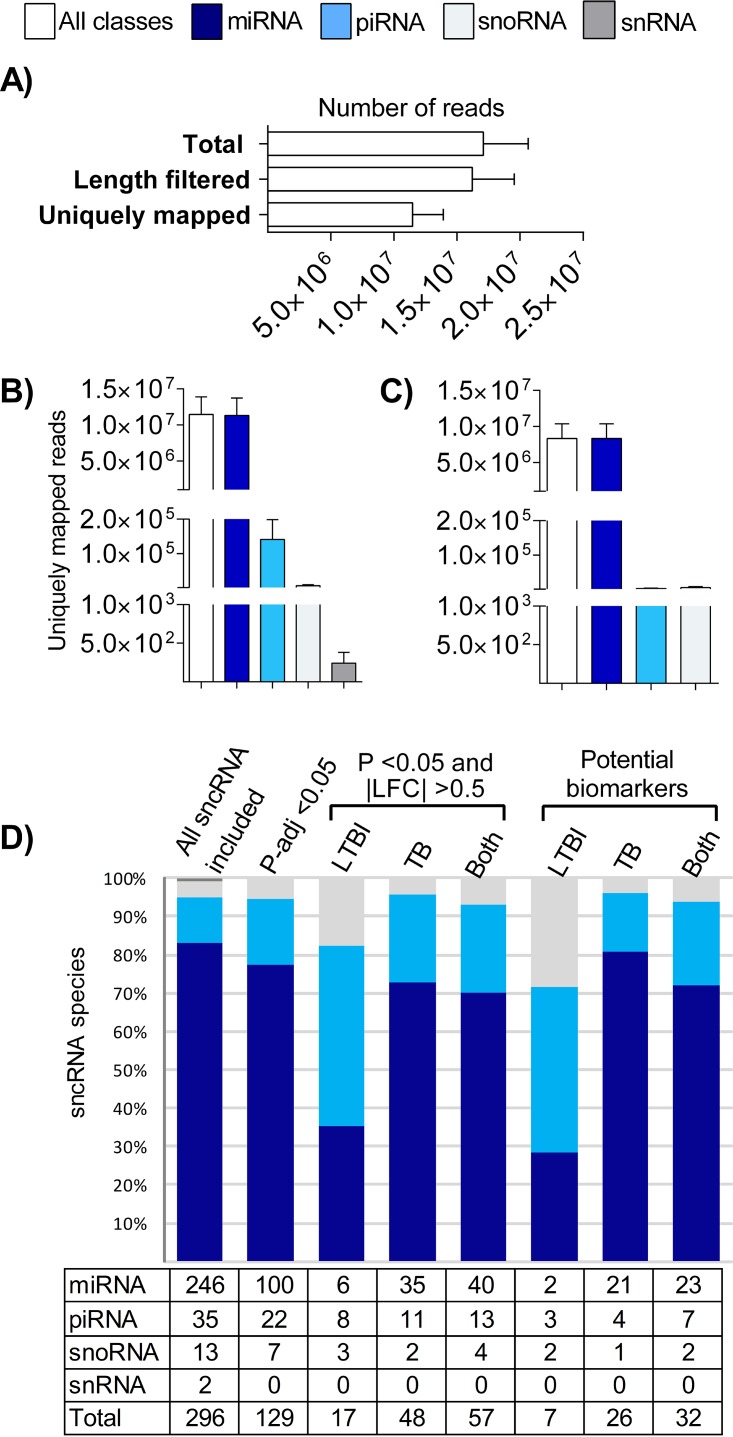
Differential representation of miRNA, piRNA, snoRNA, and snRNA in the sncRNA population before and after applying different filters. (A) Total number of sequenced reads, reads that passed the length filter (15 to 32 nt), and sncRNA uniquely mapped to the hg38 human genome. (B and C) Total number of counts of all mapped sncRNA transcripts that possessed ≥5 reads in all samples (B) and number of those that were thus included in this study after including only transcripts with a *P*-adj value of <0.05 in a multiple-group comparison (C). (D) Total numbers and percentages of miRNA, piRNA, snoRNA, and snRNA after the sequential application of the following filters: sncRNA included in panel B; sncRNA included in panel C; sncRNA differentially expressed (*P* value of <0.05 and |LFC| value of >0.5) in pairwise analyses of comparisons of LTBI to ExC and LTBItt (labeled LTBI) or of TB to LTBI, LTBItt, and ExC (labeled TB) or DE in both of these analyses (labeled Both); sncRNA fulfilling criteria for potential biomarkers in ROC curve analysis (lower 95% CI value of >0.5, asymptotic *P* value of <0.05) in the comparisons of LTBI to ExC and LTBItt (LTBI) or of TB to LTBI, LTBItt, and ExC (TB) or identified in both of these analyses (Both). The bars in panels A, B, and C correspond to means (SD).

The mapping statistics were analyzed to evaluate the abundance variation of the sncRNA species across all groups. Of the ∼17 million reads sequenced, after adaptor removal and filtering for a size range of 15 to 32 nucleotides, an average of 11.48 million (standard deviation [SD], 2.43 × 10^6^) sncRNA reads were uniquely mapped to the hg38 human genome (Fig. 1A). To reduce the possibility of bias resulting from the presence of transcripts expressed at low levels (25), we included only sncRNA with ≥5 reads in all samples. Applying this filter resulted in a minor reduction in the total number of uniquely mapped reads to 11.46 million (SD, 2.43 × 10^6^), and it also eliminated the differences in the number of mapped reads compared to snRNA (compare panel A to panel B in Fig. S1 in the supplemental material), with the result that now there were no statistical differences in the number of mapped reads in any of the four sncRNA classes across the diagnostic groups. Consistent with this, applying the filter resulted in only a slight change in variance among the diagnostic groups. This is illustrated in a principal-component analysis (PCA) plot, which also shows a high degree of overlapping of the diagnostic groups typically seen in small RNA studies of whole blood in humans (Fig. S2). Taken together, these data suggest that applying this filter eliminated mostly background signals of little biological significance. The relative composition of the remaining 296 sncRNA was roughly the same as that of the 619 sncRNA before the filter step in that miRNA constituted by far the most abundant (83.1%) class, followed by piRNA and snoRNA (Fig. 1D; see also Fig. S1B).

10.1128/mBio.01037-19.1 FIG S1 Descriptive statistics for the process of selection of sncRNA species to be included in the analysis. The bars show mean values (SD) of the total number of mapped sncRNA counts from the Oasis 2.0 web application (64) output, containing reads that were uniquely mapped to the human genome (A), and transcripts that possessed ≥5 reads in all samples and were thus selected for the subsequent analyses (B). The pie charts indicate the number and percent distribution of the sncRNA species in each sncRNA class. *, LTBItt versus TB, *P* = 0.019. Download FIG S1, TIF file, 1.8 MB. Copyright © 2019 de Araujo et al. 2019 de Araujo et al. This content is distributed under the terms of the Creative Commons Attribution 4.0 International license.

10.1128/mBio.01037-19.2 FIG S2 PCA plots based on logarithmic expression values of all 619 sncRNA. The sncRNA were mapped to the human genome using the standard pipeline of Oasis 2.0 (A) or after applying three cumulative filters as follows: ≥5 reads across all samples (*n* = 296) (B); statistical significance across multiple groups (Benjamini-Hochberg *P*-adj, <0.05) (*n* = 129) (C). (D) sncRNA differentially expressed (*P* value of <0.05 and |LFC| value of >0.5) in pairwise analyses of comparisons LTBI to ExC and LTBItt or of TB to LTBI, LTBItt, and ExC (*n* = 57). Each dot represents one sample, and diagnoses correspond to the color scheme in the legends; the circles represent 95% confidence intervals. Download FIG S2, TIF file, 2.1 MB. Copyright © 2019 de Araujo et al. 2019 de Araujo et al. This content is distributed under the terms of the Creative Commons Attribution 4.0 International license.

### Differences in sncRNA reprogramming.

**FIG 2:**
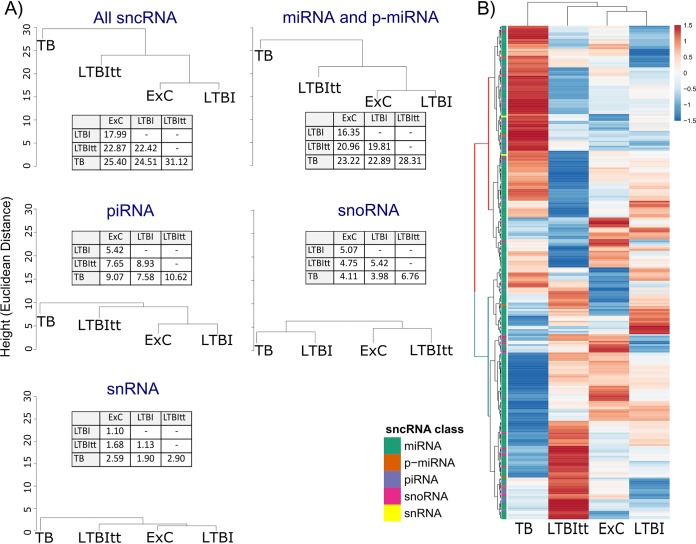
Differential reprogramming of miRNA, piRNA, snoRNA, and snRNA in the four diagnostic groups. Analysis was performed on the basis of mean expression of the 296 sncRNA possessing ≥5 reads in all samples. (A) Hierarchical clustering based on the average logarithmic values of the normalized expression data of all 296 sncRNA or only miRNA (*n* = 241 miRNAs, 5 putative miRNAs [p-miRNA]), piRNA (*n* = 35), snoRNA (*n* = 13), or snRNA (*n* = 2). The dendrograms were generated using the Euclidean distance metric; the distance values are shown in the tables. (B) Biclustering analysis based on the same 296 sncRNA as described for panel A, illustrating global expression differences and relationships among the four diagnostic groups. The four groups were clustered by Euclidean distance between rows/columns and single-linkage clustering. The normalized expression values were log transformed; the colored bar along the top of the heat map indicates the z-scores, and the color code on the left identifies each sncRNA class.

Hierarchical clustering was then used to test whether the extent of expression change of these four sncRNA classes differed among the biological groups (compared to ExC) (Fig. 2A). This analysis showed that the combined group of all sncRNA and three of the four subclasses (except snoRNA) separated active TB into its own clade from the other diagnostic groups. Indeed, active TB induced the greatest expression change with respect to ExC (as indicated by Euclidean distances; Fig. 2A) in both the global (all sncRNA) and the sncRNA class-specific analyses. snoRNA formed an exception in that they grouped the M. tuberculosis-infected groups (LTBI and TB) in one clade and that the Euclidian distance was greatest between LTBI and ExC. Consistent with the observation that the level of sncRNA reprogramming was greatest in TB, the biclustering analysis results shown in Fig. 2B revealed 2 major clades (featured in red or blue) of sncRNA which were mostly driven by expression changes in TB. However, the LTBI and LTBItt groups also displayed shared and distinct expression patterns which are addressed below in the respective differential expression (DE) analyses.

Additional criteria were then applied to the data set in order to detect sncRNA dysregulation of biological importance and statistical significance. We first selected the transcripts with a false-discovery-rate (FDR [adjusted *P* value {*P*-adj}]) of <0.05 in a multiple-group comparison (Fig. 1C). We then selected the transcripts with a *P* value of <0.05 and a |log2 fold change (LFC)| value of >0.5 in any pairwise comparison of LTBI to ExC or LTBItt or of TB to each of the other three groups (Fig. 1D). These filters resulted in the elimination of (i) all snRNA and (ii) nearly all of the most highly expressed other sncRNA (normalized expression values >50,000; also see Fig. S3), probably because the latter corresponded to constitutively expressed “housekeeping” sncRNA. Indeed, the drastic reduction in the level of the piRNA counts (compare Fig. 1B and C) was due to the removal of a single transcript (piR_016658), which has been described as the most highly expressed piRNA in whole blood and leukocytes (26).

10.1128/mBio.01037-19.3 FIG S3 Range of expression (abundance) across the four sncRNA classes. Mean normalized expression values were grouped according to an arbitrary range and to each sncRNA class. The tables indicate the number of transcripts in a given expression range, and the bars represent the respective percentages. The changes caused by successive application of three filter criteria are shown as follows: (A) ≥5 reads across all samples; (B) *P*-adj, <0.05; (C) *P* value of <0.05 and |LFC| value of >0.5. Download FIG S3, TIF file, 2.0 MB. Copyright © 2019 de Araujo et al. 2019 de Araujo et al. This content is distributed under the terms of the Creative Commons Attribution 4.0 International license.

The composition of the remaining stringently selected 57 DE sncRNA (39 miRNA, 13 piRNA, 4 snoRNA, and 1 putative miRNA [p-miRNA]) differed from that of the prefilter population in that the relative contributions of piRNA and snoRNA were now greater (Fig. 1D). A total of 17 sncRNA were classified as LTBI related, and a total of 48 were classified as TB related. piR_020490, piR_017936, piR_009059, piR_020548, piR_019912, miR-4286, miR-99b-5p, and SNORD104 were commonly found in both LTBI- and TB-related analyses, indicating that the patterns of sncRNA dysregulation of these two stages of M. tuberculosis infection may overlap. PCA based on these sncRNA revealed a further reduction in the overall variance in the sncRNA populations and a more distinct spatial distribution of the diagnostic groups (Fig. S2). The 17 LTBI-related sncRNA were predominantly downregulated as follows: 6 of 7 piRNA, 4 of 7 miRNA, and all 3 snoRNA. In active TB, DE miRNA were also predominantly downregulated (*n* = 20/35), but piRNA were predominantly upregulated (*n* = 8/11). The 2 TB-associated snoRNA were regulated in opposite directions from each other (Table S2).

10.1128/mBio.01037-19.7 TABLE S2 Number of downregulated vs. upregulated DE transcripts in the 6 pairwise analyses. Download Table S2, XLSX file, 0.01 MB. Copyright © 2019 de Araujo et al. 2019 de Araujo et al. This content is distributed under the terms of the Creative Commons Attribution 4.0 International license.

### sncRNA expression changes associated with LTBI.

**FIG 3:**
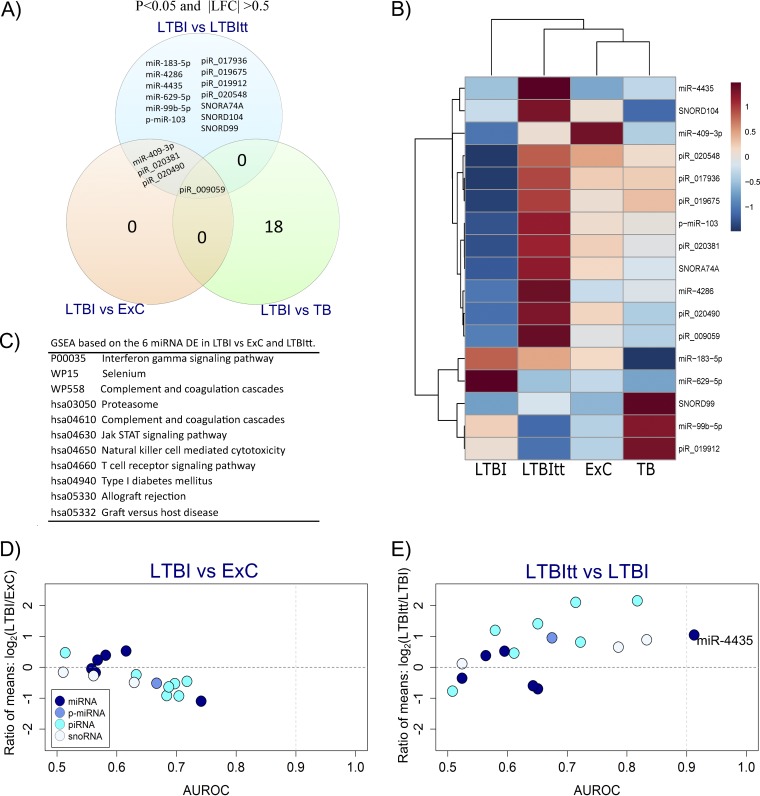
Differential sncRNA expression, gene set enrichment analysis, and biomarker screening reveal a modest degree of sncRNA reprogramming in LTBI. Analyses were performed on the basis of the 17 sncRNA that passed the filters of statistical significance (multiple group: *P*-adj, <0.05) and differential expression in the pairwise comparisons of LTBI versus ExC and LTBItt (*P* value of <0.05 and |LFC| value of >0.5). Results of analysis of the 18 sncRNA DE exclusively between LTBI and TB are shown in Fig. 4. (A) Venn diagram illustrating distinct and overlapping differential expression patterns of sncRNA in the three possible pairwise comparisons (ExC versus LTBI, *n* = 4 sncRNA; LTBI versus LTBItt, *n* = 17; LTBI versus TB, *n* = 19). (B) Heat map based on the average logarithmic values of the normalized expression data with clustering analysis of the 17 sncRNA DE in LTBI versus ExC and LTBItt. (C) Gene set enrichment analysis performed with the 6 LTBI-related miRNAs. Two (miR-183-5p and miR-409-3p) of the 6 LTBI-related miRNAs (miR-183-5p, miR-409-3p, miR-4286, miR-4435, miR-629-5p, and miR-99b-5p) were predicted to significantly modulate 11 pathways. (D and E) Biomarker screening using dispersion plots illustrating DE (ratio of means [also known as fold change], *y* axis) and biomarker potential (area under the ROC curve [AUROC], *x* axis) for the comparisons of LTBI versus ExC (D) and LTBI versus LTBItt (E). Three potential biomarkers (AUROC lower 95% CI value of >0.5, *P* value of <0.05) were identified for LTBI versus ExC and four for LTBI versus LTBItt (more-detailed information is given in Table S5). Each dot represents one sncRNA species; the corresponding sncRNA class is identified by the fill color.

The 18 sncRNA that were exclusively DE between LTBI and TB are discussed in the next section. A total of 17 sncRNA were specifically DE between LTBI and LTBItt or ExC (Fig. 3A). Four of these (miR-409-3p, piR_009059, piR_020381 and piR_020490) were DE with respect to both ExC and LTBItt, suggesting that they reflect an M. tuberculosis-uninfected state through a “normalization” of expression after isoniazid (INH) preventive treatment (IPT). The other 13 were DE uniquely between LTBI and LTBItt; only 1 of them was also DE between LTBItt and ExC (Fig. S5), suggesting that the remaining 12 do not reflect adverse effects of IPT (e.g., persisting drug toxicity) but clearance of the infection. piR_009059 was unique in that its expression was significantly lower in LTBI than in the other three groups.

10.1128/mBio.01037-19.5 FIG S5 SncRNA differentially expressed in LTBItt with respect to LTBI and ExC. Analyses were performed on the basis of the 57 sncRNA that passed the filters of statistical significance (multiple group; *P*-adj, <0.05) and of the magnitude of the difference between means (pairwise comparisons against LTBItt; *P* < 0.05 and |LFC| value of >0.5). (A) Venn diagram illustrating distinct and overlapping differential expression patterns in the pairwise comparisons of LTBItt to ExC and LTBI. (B) Heat map based on the average logarithmic values of the normalized expression data with clustering analysis of the 18 sncRNA DE in LTBItt (ExC versus LTBItt, *n* = 1; LTBItt versus LTBI, *n* = 18). Download FIG S5, TIF file, 2.1 MB. Copyright © 2019 de Araujo et al. 2019 de Araujo et al. This content is distributed under the terms of the Creative Commons Attribution 4.0 International license.

10.1128/mBio.01037-19.10 TABLE S5 Results of biomarker evaluation using receiver operating characteristic (ROC) curve analysis. AUROC values, 95% confidence interval (95% CI) and asymptotic P values for binary (pairwise) ROC analysis. (A) 17 LTBI-related sncRNA (LTBI vs ExC or LTBItt). (B) 48 TB-related sncRNA (TB vs LTBI, ExC or LTBItt). Potential biomarker candidates (as defined by a lower limit 95% CI > 0.50 and asymptotic P values of the ROC curve <0.05) are identified by light grey shading; their total number (n) and their percentage (%) of all sncRNA in the respective comparisons are shown in the pie charts. The members of the 4-scnRNA component of the proposed TB-classifier (see Fig. 5) are identified in bold. Download Table S5, XLSX file, 1.1 MB. Copyright © 2019 de Araujo et al. 2019 de Araujo et al. This content is distributed under the terms of the Creative Commons Attribution 4.0 International license.

Gene set enrichment analysis (GSEA) was performed in order to identify biological pathways potentially regulated by miRNA in LTBI. It revealed that 2 of the 6 LTBI-related miRNA, i.e., miR-183-5p (which was DE only with respect to LTBItt) and miR-409-3p (DE in LTBI versus both ExC and LTBItt) were significantly associated with the IFN-γ signaling pathway, as well as with other pathways functionally related to immune responses to M. tuberculosis (Fig. 3C; see also Table S4). Considering that the IGRA is based on IFN-γ responses and that the TST can also reflect IFN-γ responses (27), we performed an analysis of correlation between all LTBI-related sncRNA and the quantitative responses to these LTBI screening tests across the 14 ExC plus 21 LTBI samples together (*n* = 35). Indeed, significant correlations were observed for both miRNAs; miR-183-5p correlated positively with st-IGRA (*P* = 0.02), and miR-409-3p correlated negatively with TST (*P* = 0.016) and lt-IGRA (*P* = 0.003) (Table 2). In addition, piR_017936 (*P* = 0.01) and miR-4286 (*P* = 0.033) correlated negatively with st-IGRA, whereas neither the other sncRNA nor the presumably stably expressed internal control RNU6P-233P (28) correlated significantly with any of the LTBI tests.

**TABLE 2:** Correlations between sncRNA expression and reactivity to TST and short-term and long-term IGRA^*a*^

Assay or transcript	TST (mm)^*b*^	st-IGRA (pg/ml)	lt-IGRA (pg/ml)
st-IGRA	0.56***		
lt-IGRA	0.66***	0.54***	
piR_009059	−0.25	−0.18	−0.28
piR_017936	−0.17	−0.42**	−0.18
piR_019675	−0.26	−0.29	−0.23
piR_019912	−0.12	−0.024	−0.091
piR_020381	−0.24	−0.28	−0.24
piR_020490	−0.21	−0.23	−0.33
piR_020548	−0.12	−0.27	−0.18
miR-183-5p	0.01	0.38*	0.06
miR-409-3p	−0.41*	−0.16	−0.48***
miR-4286	−0.09	−0.36*	0.034
miR-4435	0.11	0.11	0.001
miR-629-5p	0.11	0.27	0.13
miR-99b-5p	−0.03	0.05	0.01
p-miR-103	0.02	−0.03	0.01
SNORA74A	−0.13	−0.08	−0.19
SNORD99	0.08	0.01	0.01
SNORD104	−0.04	0.17	−0.25
RNU6-223P	0.12	0.05	0.11

a lt, long term; st, short term; *, *P* < 0.05; **, *P* < 0.01; ***, *P* < 0.005.

b Diameter of induration.

10.1128/mBio.01037-19.9 TABLE S4 Gene set enrichment analysis performed with the 7 miRNA (miR-99b-5p, miR-183-5p, miR-185-5p, miR-409-3p, miR-629-5p, miR-4286, and miR-4435) DE in pairwise comparions between LTBItt vs. ExC and LTBI. Download Table S4, XLSX file, 0.01 MB. Copyright © 2019 de Araujo et al. 2019 de Araujo et al. This content is distributed under the terms of the Creative Commons Attribution 4.0 International license.

Seven LTBI-related sncRNA qualified as potential biomarkers (lower confidence interval [CI] value of >0.5 and asymptotic *P* value of <0.05 in receiver operating characteristic [ROC] analysis) to discriminate LTBI from ExC (miR-409-3p, piR_017936, and piR_019675) or from LTBItt (miR-4435, piR_009059, SNORA74A, and SNORD104) (Table S5). In this subset, the overall distribution of the sncRNA classes had changed from the original profile (Fig. 1D; see also Table S5), as it now contained a much larger fraction of piRNA. The highest area under the ROC curve (AUROC) values for the distinction between LTBI and ExC were 0.74 (miR-409-3p) and 0.72 (piR_017936), revealing only moderate biomarker potential for sncRNA to identify LTBI among ExC. On the other hand, upregulation of miR-4435 constituted a highly accurate biomarker candidate (AUROC = 0.91) for accomplished IPT (Fig. 3D and E).

### sncRNA expression changes associated with active TB.

**FIG 4:**
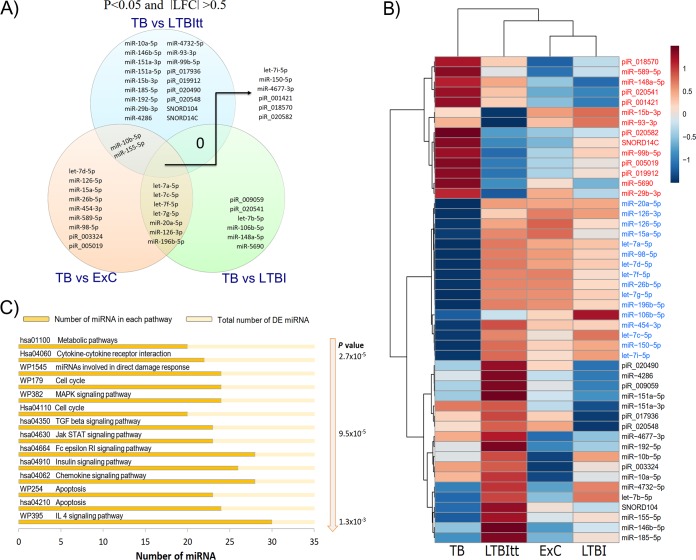
TB-related differential expression and gene set enrichment analysis reveal a strong impact of active disease on sncRNA reprogramming. Analyses were performed on the basis of the 48 sncRNA that passed the filters of statistical significance (multiple group: *P*-adj, <0.05) and differential expression in the pairwise comparisons of TB versus LTBI, LTBItt, and ExC (*P* value of <0.05 and |LFC| value of >0.5). (A) Venn diagram illustrating distinct and overlapping differential expression patterns in the three possible pairwise comparisons (ExC versus TB, *n* = 24 sncRNA; LTBI versus TB, *n* = 19; LTBItt versus TB, *n* = 26). (B) Heat map based on the average logarithmic values of the normalized expression data with clustering analysis of the 48 sncRNA DE in TB. (C) Gene set enrichment analysis based on the 35 TB-related miRNA. A total of 104 pathways were significantly enriched, and the 15 with the lowest *P* values (*P* ≤ 1.4 × 10^−3^) are shown. IL 4, interleukin 4.

48 sncRNA (35 miRNA, 11 piRNA, and 2 snoRNA; Fig. 4A) were DE between the active TB group and the other 3 groups. Six sncRNA (let-7i-5p, miR-150-5p, miR-4677-3p, piR_001421, piR_018570, and piR_020582) were globally associated with active TB, as they were DE in all comparisons involving TB (Fig. 4A, center). Other transcripts were DE only in comparison with two specific groups; 7 miRNAs (containing four members of the let-7 family) were DE versus ExC and LTBI, 2 miRNAs (miR-10b-5p and miR-155-5p) versus ExC and LTBItt, and 18 sncRNA (also including 4 piRNAs and 2 SNORDs) only versus LTBItt. The latter result suggested that changes in sncRNA expression may reveal biomarkers to detect progression to TB after IPT (Fig. 4A).

Consistent with the global clustering analysis shown in Fig. 2B, hierarchical clustering based on the subset of 48 TB-related sncRNA placed the TB group in its own clade, and 2 major clades of transcripts that were upregulated (*n* = 14; labels in red font) or downregulated (*n* = 16; blue font) in TB were evident (Fig. 4B). Interestingly, expression of a set of miRNAs from the let-7 family (let-7a-5p, let-7b-5p, let-7c-5p, let-7d-5p, let-7f-5p, let-7g-5p, and let-7i-5p) was significantly decreased in TB. A third clade (Fig. 4B, black font; *n* = 18) was predominantly, but not exclusively, composed of transcripts DE only between LTBItt and TB (*n* = 12/18).

GSEA based on the 35 TB-related miRNA revealed enrichment of 104 pathways (Table S3). The subcategories with the highest significance values (*P* ≤ 0.00014) corresponded to pathways modulating metabolism and mitogen-activated protein kinase (MAPK), transforming growth factor β (TGF-β), JAK-STAT, Fc epsilon RI, insulin, interleukin, and presenilin signaling, as well as cytokine-cytokine receptor interactions, direct-damage responses (DDR), cell cycle, and DNA damage (Fig. 4C). Of note, 23 of these 35 miRNAs were predicted to affect pathways associated with B lymphocytes (CD19^+^ cells).

10.1128/mBio.01037-19.8 TABLE S3 Gene set enrichment analysis performed with the set of 35 miRNA DE in pairwise comparisons between TB and the other 3 diagnostic groups. Download Table S3, XLSX file, 0.02 MB. Copyright © 2019 de Araujo et al. 2019 de Araujo et al. This content is distributed under the terms of the Creative Commons Attribution 4.0 International license.

**FIG 5:**
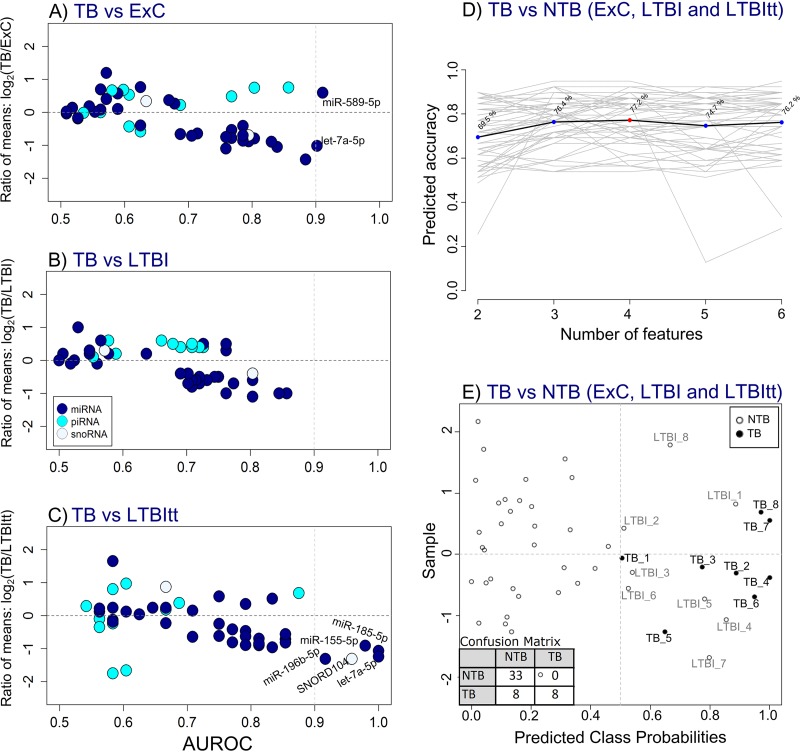
Identification of a 4-sncRNA biosignature for TB. (A to C) Biomarker screening using dispersion plots showing ratio of means and AUROC for the paired comparisons of TB versus ExC (A), LTBI (B), and LTBItt (C). Each dot represents one sncRNA species, the corresponding sncRNA class is identified by the fill color. sncRNA with high discriminatory potential (AUROC value of ≥0.90, vertical dotted line) in the comparisons between TB versus ExC and LTBItt were selected for a multivariate ROC curve analysis (shown in Fig. 5D; see also Fig. S4). (D) Plot of the predictive accuracy determined using support vector machine (SVM) models with increasing numbers of features. The most accurate biomarker combination model consisted of 4 sncRNA (highlighted in red). (E) The 4 features (let-7a-5p, miR-196b-5p, miR-589-5p, and SNORD104) with the highest selection frequency in the SVM models (Fig. S4) were included in a predictive class probability analysis to classify samples as TB or non-TB (NTB). The classification boundary for TB is at the center of the *x* axis (*x* = 0.5, vertical dotted-line), values of >0.5 classify a sample as TB, and values closer to 1 indicate a greater probability. Each dot represents an average prediction of one sample after cross-validations, along with the confusion matrix obtained. Samples are distributed along the y axis by balanced subsampling.

ROC analysis revealed a substantially higher number of potential biomarkers for active TB than for LTBI in that 26 sncRNA (21 miRNA, 4 piRNA, and 1 snoRNA; Fig. 1D; see also Table S5) were identified as potential biomarkers. A much greater contribution of miRNA (81.5%) followed by piRNA (14.8%) and snoRNA (3.7%) was observed, which was similar to the initial distribution of sncRNA classes (Fig. 1D). Of these, six sncRNA were highly accurate markers (AUROC value of >0.9) in the differentiation between the TB group and the non-TB (NTB) groups (Fig. 5A and C). miR-589-5p [AUROC = 0.91] and let-7a-5p [AUROC = 0.90] distinguished between TB and ExC and let-7a-5p [AUROC = 1.0], miR-185-5p [AUROC = 1.0], miR-155-5p [AUROC = 0.98], SNORD104 [AUROC = 0.96], and miR-196b-5p [AUROC = 0.92] between TB and LTBItt. In contrast, there was no single highly accurate biomarker for the discrimination between TB and LTBI (Fig. 5B), as the highest AUROC values were 0.86 (let-7a-5p) and 0.85 (miR-196b-5p).

10.1128/mBio.01037-19.4 FIG S4 Plot of the features most frequently selected in a support vector machine model containing 4 sncRNA. The top 4 (SNORD104, hsa-miR-196b-5p, hsa-let-7a-5p, and hsa-miR-589-5p) were selected to compose the proposed TB classifier. Download FIG S4, TIF file, 1.9 MB. Copyright © 2019 de Araujo et al. 2019 de Araujo et al. This content is distributed under the terms of the Creative Commons Attribution 4.0 International license.

### Identification of a 4-sncRNA biosignature for TB.

Considering the absence of a single accurate biomarker for the distinction between TB and LTBI, a predictive accuracy analysis based on support vector machine (SVM) models was then used to test whether a combination of sncRNA would yield a more accurate classifier that could differentiate TB from all NTB cases, including LTBI cases. The highest predicted accuracy (77.2%) was observed for a classifier consisting of the top 4 sncRNA with the highest selection frequencies by the SVM models (let-7a-5p, miR-196b-5p, miR-589-5p, and SNORD104; Fig. S4), which was subsequently evaluated in a predictive class probability analysis involving all four diagnostic groups (Fig. 5E). This classifier identified 16 individuals as TB. Their main clinical and laboratory data are summarized in Table 3. Eight of these corresponded to the correctly classified 8 TB cases included in this study (100% sensitivity), but another 8 stemmed from the LTBI group (Fig. 5E). Of note, 5/8 (62.5%) of these reclassified LTBI (LTBI^reclas^) cases had abnormalities on CXR that could be attributed to M. tuberculosis infection (29) (Table 3). Importantly, none of the ExC or LTBItt cases were classified as TB. In fact, the LTBItt group had the lowest class probability (average, 6.2% [SD, 6.5]), followed by ExC (17.7% [SD, 14.5]) and LTBI (38.7% [SD, 26.8]). The mean class probability value determined for the TB group was 84.2% (SD, 17.2), and the lowest predicted probability was observed for the one patient with a normal CXR and negative AFB stain and sputum culture, harboring a pleural TB infection (TB_1 = 50.5%; Table 3).

**TABLE 3:** Characteristics of the 16 subjects and their class probabilities predicted by the TB classifier based on the expression values of let-7a-5p, miR-196b-5p, miR-589-5p, and SNORD104 in whole blood^*a*^

Sample	Patient age (yrs)	Sex	CXR result		TST (mm)^*b*^	Sputum smear result	Culture result	Examination result						Original diagnosis	TB classifier- predicted class probability (%)
			Medical report	Probabilityof an *M. tuberculosis* infection				Cough	Hemoptysis	Fever	Wt loss	Dyspnea	Chest pain		
LTBI_1	63	M	Parenchymal TB scar	High	15	−	−	+	−	−	+	+	+	LTBI	88.6
LTBI_2	48	F	Normal	No	15	ND	ND	−	−	−	−	−	−	LTBI	51.1
LTBI_3	49	F	Normal	No	16	ND	ND	+	−	−	−	−	−	LTBI	53.9
LTBI_4	40	M	Calcified scar near the hilum	Possible	10	−	−	−	−	−	−	−	−	LTBI	85.4
LTBI_5	43	F	Parenchymal TB scar	High	15	−	−	+	−	−	−	−	−	LTBI	78.1
LTBI_6	38	M	Normal	No	14	ND	ND	−	−	−	−	−	−	LTBI	52.6
LTBI_7	48	F	Parenchymal TB scar	High	15	−	−	−	−	−	−	−	−	LTBI	79.7
LTBI_8	68	M	Basal parenchyma thickening	Possible	6	−	−	+	−	−	−	−	−	LTBI	66.6

TB_1	28	M	Normal	No	0	−	+¥	−	−	+	+	+	+	Pleural TB	50.5
TB_2	30	F	Cavitary TB	High	12	−	+	−	−	−	−	−	−	TB	88.7
TB_3	55	M	Cavitary TB	High	8	+	+	+	−	+	+	−	+	TB	77.4
TB_4	72	M	Nodules (4–6 cm) in the apical area, emphysema, and swollen lymph notes	Possible	8	−	+	+	+	+	+	+	−	TB	100
TB_5	49	F	Noncavitary TB	High	ND	+	+	+	−	+	+	−	−	TB	64.9
TB_6	18	M	Cavitary TB	High	8	+	+	+	−	+	+	−	+	TB	94.9
TB_7	65	F	Noncavitary TB/ parenchymal TB scar	High	15	−	+	+	−	−	+	−	−	TB	100
TB_8	59	M	Noncavitary TB	High	10	+	+	−	−	−	−	−	−	TB	97.1

a Abbreviations: AFB, acid-fast bacilli; CXR, chest X-ray; F, female; M, male; ND, not done; ¥, culture of a pleural tissue sample.

b Diameter of induration.

**FIG 6:**
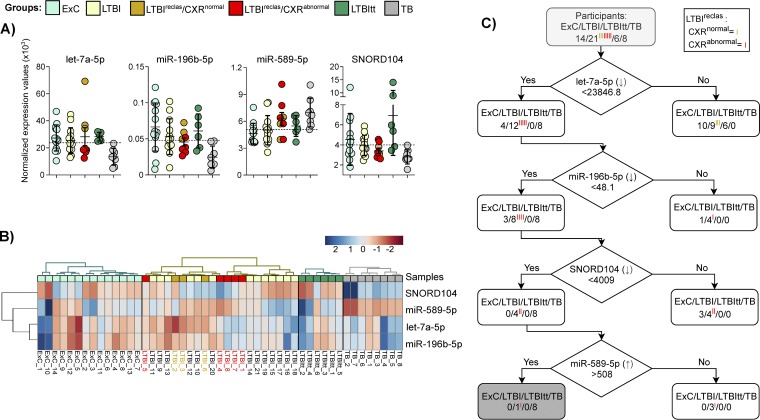
Heterogeneity in sncRNA expression supports the use of combinations of biomarkers to improve accuracy for TB detection. An expression profile of the 4 sncRNA (let-7a-5p, miR-196b-5p, miR-589-5p, and SNORD104) constituting the proposed TB classifier identified in Fig. 5E (see also Fig. S4) is illustrated. (A) Dot plots based on the normalized expression values (the same data are used for Fig. 5A and for panel C of this figure). The solid horizontal lines represent the median; the dotted lines represent the cutoff value used for the data shown in panel C. (B) Heat map created using normalized sncRNA expression values that were median centered, log transformed, and scaled by the Pareto method in order to facilitate visual comparability among expression profiles with different expression ranges. The dendrogram was generated by hierarchical clustering analysis by sncRNA species, with group assignments held constant. The colored bar along the top of the heat map indicates the z-scores. (C) Decision tree analysis based on let-7a-5p, miR-196b-5p, miR-589-5p, and SNORD104. Sensitivity of 100% was achieved for TB by let-7a-5p alone, and sequential addition of the other three sncRNA led to increasing specificity (maximum = 97.6%). The arrows indicate the direction of the transcription modulation during active TB.

Individual normalized counts of the components of this 4-sncRNA TB classifier in the four diagnostic groups are shown in Fig. 6A, followed by a hierarchical clustering analysis (Fig. 6B). This analysis revealed that the three miRNAs were contained in one clade, suggesting common regulatory features that were distinct from regulation of SNORD104 (Fig. 6A). Both panel A and panel B of Fig. 6 clearly show the heterogeneity in expression in the NTB subgroups, which agrees well with the reported results (Fig. 5D) indicating that a combination of several biomarkers was necessary to achieve clinically relevant accuracy of TB detection.

### Classification tree analysis.

To assess the contribution of each of the four members of the sncRNA classifier, we performed a classification tree analysis comprising all 49 samples (Fig. 6C). Whereas 100% sensitivity for TB was achieved by let-7a-5p alone, adding each of the other 3 sncRNA resulted in increasing specificity, which reached a final value of 97.6% in the last branch, i.e., after addition of miR-589-5p. Of note, the three CXR-negative LTBI^reclas^ subjects were classified as non-TB already in the first step, suggesting that they share an sncRNA pattern which is not as strongly suggestive of TB as that of the other 5 LTBI^reclas^.

### Additional transcriptomic evidence of advanced LTBI in LTBI^reclas^ subjects with abnormal CXR.

**FIG 7:**
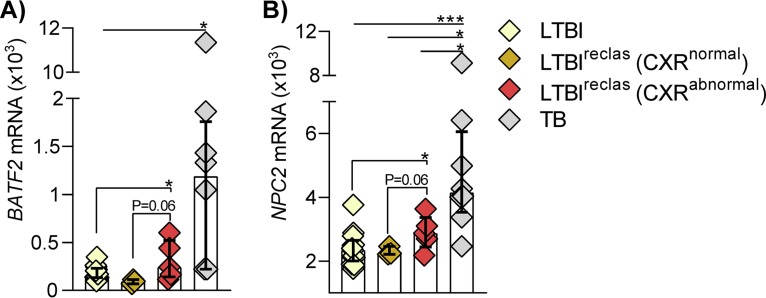
Expression of *BATF2* (A) and *NPC2* (B) mRNA suggests an elevated risk of progression to TB in LTBI^reclas^ with abnormal chest radiograph. Dot plots based on the normalized expression values indicate that expression in the CXR-abnormal LTBI^reclas^ subjects (*n* = 5) tends to be intermediate between LTBI (*n* = 13, classified as NTB by logistic regression [Fig. 5E]) and TB (*n* = 8). *y* axis = normalized mRNA expression values in whole blood. *P* values: *, <0.05; ***, <0.001 (T-test, one-tailed).

Since mRNA expression data were available for the same samples, we then tested whether the 4-sncRNA classifier could be validated by two established mRNA biomarkers for TB, *NPC2* (30) and *BATF2* (31). Expression of both mRNAs was higher in the CXR-abnormal LTBI^reclas^ subjects than in the LTBI subjects that had been classified as LTBI by logistic regression (Fig. 5E, yellow symbols; *n* = 13; *P* value of <0.05), and it also tended to be higher (*P* = 0.06) than in the three CXR-negative LTBI^reclas^ subjects (Fig. 7). In addition, *BATF2* expression levels did not differ significantly between the CXR-abnormal LTBI^reclas^ and the 8 TB cases. Taken together, these results indicate that the 5 CXR-abnormal LTBI^reclas^ subjects have transcriptomic features suggestive of an intermediate state between latent and active TB.

### Transcriptional profiles of let-7a-5p, miR-196b-5p, miR-589-5p, and SNORD104 during progression to active TB and after successful treatment.

**FIG 8:**
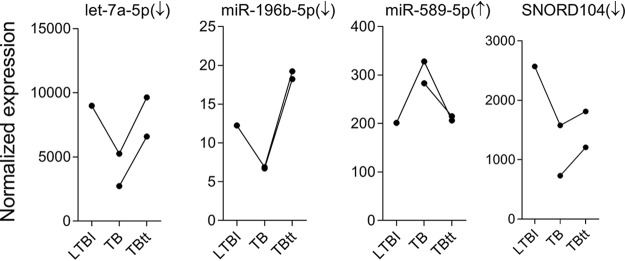
Temporal changes in the levels of expression of let-7a-5p, miR-196b-5p, miR-589-5p, and SNORD104 indicate a trend of normalization after completed anti-TB therapy in two TB cases. Two rCt progressed to active pulmonary TB during this study. Follow-up blood samples were additionally collected as follows: during LTBI (*n* = 1), upon progression to active pulmonary TB (*n* = 2), and after anti-TB treatment (TBtt; *n* = 2). The raw reads from these samples were normalized following the same methods and standards used in the cross-sectional study. The arrows indicate the direction of the transcription modulation during active TB.

To test from a different angle whether DE of the 4 components of the sncRNA classifier was associated with active disease, their expression levels were plotted for the two TB patients from whom a blood sample was available after completed treatment. In both patients, expression of all four sncRNA tended to normalize after treatment (Fig. 8). Of note, for one case we also had a sample obtained during LTBI, and the expression change that had occurred after progression to TB was in the expected direction for all members of the classifier, i.e., downregulation for let-7a-5p, miR-196b-5p, and SNORD104, but upregulation for miR-589-5p.

### Similar expression profiles of let-7a-5p, miR-196b-5p, miR-589-5p, and SNORD104 in peripheral whole blood in TB and in M. tuberculosis-infected PBMC.

**FIG 9:**
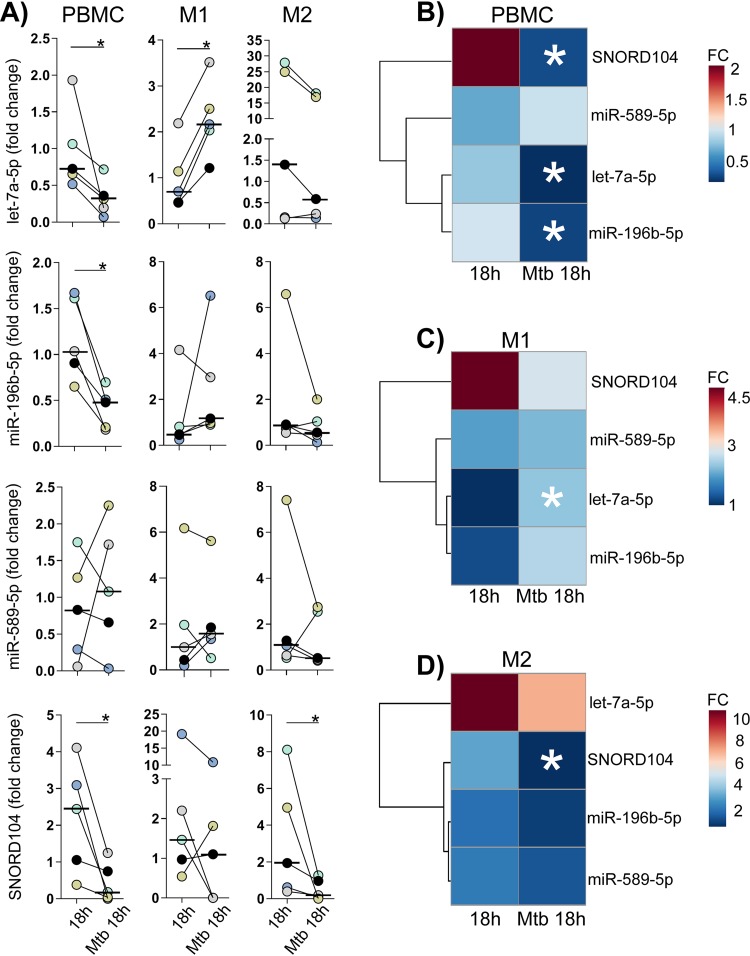
M. tuberculosis-induced expression changes of the components of the 4-scnRNA classifier in peripheral blood mononuclear cells resemble those seen in whole blood of TB cases. Relative let-7a-5p, miR-196b-5p, miR-589-5p, and SNORD104 expression levels were evaluated in peripheral blood mononuclear cells (PBMC) and in blood-derived M1 and M2 macrophages (*n* = 5 donors) after 18 h of infection with M. tuberculosis H37Rv or mock infection. U6 was used as an internal control and was stably expressed across all samples (Friedman test, *P* = 0.94). Fold change was calculated using *t* = 0 h as reference. *, Wilcoxon matched-pair signed rank test *P* value of <0.05. (A) Dot plots with the individual fold changes, a different color was assigned to each donor. Small bars = median. (B to D) Heat maps based on mean fold change (FC) values and row clustering of M. tuberculosis-infected PBMC (B) and M1 (C) and M2 (D) macrophages. The colored bars along the top of the heat map indicate mean FC values. As in whole blood from TB cases (see Fig. 6A and B), let-7a-5p, miR-196b-5p, and SNORD104 were downregulated in PBMC, whereas in macrophages only expression of SNORD104 (M2) changed in the same direction as in whole blood.

To test whether the observed changes in expression of let-7a-5p, miR-196b-5p, miR-589-5p, and SNORD104 in TB (Fig. 4B and 5C) could be related to infection of human host cells by M. tuberculosis (or to exposure of the cells to the pathogen or its antigens), we measured their expression levels in a cellular infection model using primary human peripheral blood mononuclear cells (PBMC) and M1-type and M2-type macrophages (Fig. 9). Compared to DE in TB (e.g., Fig. 6A), expression changes were most similar in infection of PBMC in that SNORD104, let-7a-5p, and miR-196b-5p were all downregulated and that a slight tendency of miR-589-5p upregulation was observed (Fig. 9A). In addition, the three miRNA clustered together in one clade, as had been observed in peripheral blood (compare Fig. 9B and 6B). Expression changes in differentiated macrophages agreed only in terms of significant downregulation of SNORD104 in M2 and a tendency toward downregulation of SNORD104 in M1. Let-7a-5p was downregulated in PBMC and upregulated in M1 macrophages but was not DE in M2 macrophages (Fig. 9A), indicating that its regulation patterns may differ by cell type. Taken together, these results demonstrate that, compared to whole blood (from which the sncRNA expression profiles of the study cohort had been obtained), expression of the 4-sncRNA classifier was most similar in PBMC, i.e., in the blood fraction most closely related to whole blood.

### External validation.

Two previously published microarray-based miRNA data sets were available for external validation of the three miRNA contained in our 4 sncRNA TB classifier. These studies were conducted in Gambia (GEO data set GSE39163) and Germany (GSE34608). Let-7a-5p (AUROC value = 1.0) demonstrated excellent discrimination between HD and TB among Europeans (GSE34608), while miR-196b-5p and miR-589-5p performed better in the Gambian population (GSE39163) but with lower AUROC values (0.67 and 0.73) (Table 4). All these miRNA showed expression changes in the same direction as in the Brazilian population.

**TABLE 4:** Validation of the 3 miRNA components of the proposed classifier for active TB by reanalysis of two external studies^*a*^

miRNA component	Present study (Brazil)				GSE34608 study (Germany)		GSE39163 study (Gambia)	
	AUROC ExC (*n* = 14) vs TB (*n* = 8)	Direction of regulation	AUROC LTBI (*n* = 21) vs TB (*n* = 8)	Direction of regulation	AUROC HD (*n* = 8) vs TB (*n* = 8)	Direction of regulation	AUROC LTBI (*n* = 8) vs TB (*n* = 8)	Direction of regulation
let-7a-5p	0.90	↓	0.86	↓	1.0	↓	0.52	—
miR-196b-5p	0.88	↓	0.85	↓	0.56	—	0.67	↓
miR-589-5p	0.91	↑	0.67	↑	0.63	↑	0.73	↑

a Symbols: ↓ = downregulation; ↑ = upregulation. Abbreviations: HD = healthy donor; ExC = exposed control; LTBI = latent TB infection; TB = tuberculosis; AUROC = area under the ROC curve; — = no differential expression.

## DISCUSSION

We have conducted the first comprehensive, integrative small RNAseq analysis of peripheral blood samples from individuals with different stages of M. tuberculosis infection and compared the relative biomarker potential of the four major sncRNA classes, i.e., miRNA, piRNA, snoRNA, and snRNA. The final population of potential biomarkers contained somewhat lower contributions from miRNA (71.9% in the biomarker data set versus in the original data set [Fig. 1D; see also Table S5 in the supplemental material]) and higher contributions from piRNA (21.9% versus 11.8%) and snoRNA (6.3% versus 4.4%) than the original population of detected sncRNA but contained no snRNA. While it has been known for some time that expression and activity patterns of the noncoding RNAome (sncRNAome) exhibit considerable plasticity in biology and disease, our study was the first to formally assess the biomarker potential of the four major sncRNA classes in an important human infectious disease, and the results reveal the previously underappreciated potential of piRNA and snoRNA as biomarkers for patient classification and (pending a better understanding of their physiological functions) as tools for new insights into pathophysiological mechanisms.

SNORD104, also known as U104, is predicted to direct 2′-O-methylation to 28S rRNA (32). The 2′-O-methylation process induces ribosomal heterogeneity, mainly by driving protection from hydrolysis and modification of strand flexibility (33). Monaco et al. (33) proposed that changes in the expression levels of subclass C/D snoRNAs (SNORD104 is included in this subclass) “likely” play the role of a regulatory mechanism in the translation of specific mRNAs, due to the induction of more “specialized ribosomes” (34). Pathophysiologically relevant changes in host snoRNA expression have been observed in cells and human body fluids during cancer and viral infections (reviewed in reference 21). However, snoRNA regulation has thus far hardly been explored in bacterial infections; together with the current study, SNORD104 dysregulation has been described previously in an investigation of another important mycobacterial infection. Its downregulation was observed in the blood of leprosy patients suffering from reversal reactions (35), representing a pathological immune reaction with granulomatous inflammation in which T-cell-mediated immunity is increased and the disease presentation shifts toward the tuberculoid pole (36). Recent evidence has additionally indicated that many snoRNA (including SNORD104) are cleaved into shorter (∼22-nt) “miRNA-like” functional forms (reviewed in reference 37). The SNORD104-derived miRNAs are highly abundant in activated mature lymphocytes and were previously reported to target ribosomal protein S3 (RPS3), an essential NF-κB binding partner (38). We have detected its downregulation upon M. tuberculosis infection in blood-derived PBMC and in M2 macrophages but not M1 macrophages. M1 phenotypes are described to exhibit better bactericidal activity and to promote granuloma formation, while M2 phenotypes would inhibit these processes (39). These results indicate that the downregulation of SNORD104 observed in peripheral blood in our study is biologically relevant and suggest that its role (or that of the miRNAs derived from it) in TB pathogenesis merits further investigations. piRNAs were DE in both LTBI and TB, and piR00905 and piR_018570 had AUROC values of >0.8 (“excellent classification” according to a common scoring scheme [40]) for differentiation between LTBI and LTBItt and between TB and ExC, respectively. Among other functions, complexes of piRNA with PIWI proteins have been implicated in locus-specific DNA methylation processes (41). Epigenetic changes in host cells caused by M. tuberculosis infection have been suggested to be a possible bacterial evasion mechanism (42, 43), which might explain the different piRNA signatures identified here. Another plausible explanation is that piRNA are simply dysregulated upon cellular stress (44). However, very little is known about the full spectrum of piRNA functions beyond the original concept that they interfere with replication of endogenous retroviruses in germ line cells. It is possible that, similarly to miRNA, they regulate a broad spectrum of biological processes, and dedicated in-depth studies on their role in M. tuberculosis infection, as well as in infectious diseases in general, will, therefore, be of great interest.

### Does sncRNA expression identify a subgroup of LTBI cases?

During latency, the site of infection is relatively shielded from the peripheral circulation and M. tuberculosis is considered less metabolically active (45). It was therefore not unexpected that we identified only three biomarker candidates for the detection of LTBI versus ExC, all of which had only moderate discriminatory potential (Table S5). Remarkably, expression of the LTBI-related miR-409-3p transcript correlated negatively with TST and IGRA responses and could be related to the IFN-γ response pathway, which is pivotal in the control of M. tuberculosis infection (46). This suggests that, even though miR-409-3p downregulation was not seen in all LTBI subjects, it may be pathophysiologically important in a subgroup whose clinical characteristics (e.g., ability to contain or resolve latent infection) remain to be defined. Although there are no other studies on sncRNA as biomarkers for LTBI, our data raise the possibility that the blood sncRNA pool may not be the optimal source for biomarkers for use in screening exposed populations for latently infected individuals *per se* and may be more useful for characterizing subpopulations.

### What is the significance of the IPT-associated sncRNA?

The inclusion of individuals who have completed IPT is another unique feature of our study. In the set of sncRNA that were DE in LTBItt, only a single one was DE with respect to ExC, suggesting that several months after completion of IPT there was little if any evidence of changes in the sncRNAome that might reflect direct effects of the INH treatment. Rather, the data suggest that the sncRNA that were DE with respect to LTBItt more likely represent markers for the immune reaction accompanying resolution of the latent infection. Of note, all 4 members of the TB signature constitute highly accurate individual biomarkers for the differentiation of TB versus LTBItt, suggesting that they may prove useful in detecting progression to TB after IPT.

### Implications of the 4-sncRNA classifier.

Analysis of our 4-sncRNA (let-7a-5p, miR-196b-5p, miR-589-5p, and SNORD104) biosignature for TB led to “reclassification” of 8 LTBI individuals as TB cases. Five of them were found to show clinical/imaging characteristics of progression to TB, and their “TB-like” features were further substantiated by increased expression of previously validated mRNA biomarkers for TB. South African individuals detected with subclinical TB (as defined by a positive IGRA associated with imaging abnormalities in the lung parenchyma such as infiltrates, fibrotic scars, and nodules) were found to be significantly more likely to develop symptomatic active TB in a 6-month prospective study (47). Considering that the infected subjects may present with different features of the disease (the so-called spectrum of TB [45]) during progression from latent to active TB, individuals identified by our classifier could then be given additional treatment and/or enrolled in more-intense follow-up programs. All 8 potential TB cases detected among LTBI patients by our classifier were subjected to IPT, and a longer-term follow-up was not envisaged in the study protocol. It was, therefore, not possible to ascertain whether any of them progressed to TB. It remains to be tested whether this 4-sncRNA classifier can be replicated in larger prospective trials focusing on progression from LTBI to TB, which should also enroll subjects with different genetic backgrounds and other pulmonary pathologies as disease controls. Considering the current limitations in detecting subgroups within the heterogeneous spectrum of TB, our report highlights that the observed reprogramming of sncRNA populations yielded small RNA biomarkers that can be used to identify difficult-to-diagnose TB cases regardless of level of lung injury (as seen on CXR images), bacterial load, or clinical symptoms. How practical would it be to implement this or a similar classifier(s) in clinical practice? Its sensitivity of 100% and specificity of 97% exceed the requirements of the World Health Organization target product profile for community-based diagnostic tests to identify individuals at risk of active TB (sensitivity of >95% and specificity of >80% [48]). Currently, the required technology (and thus the expected cost) would likely restrict its use to those medical facilities with a relatively high level of technology. However, this scenario might change in the near future with the development of more cost-effective point-of-care technologies for RNA amplification (49).

### Value of the external validation.

We validated the miRNA components of the classifier by interrogating two data sets from different ethnicities. In particular, let-7a-5p turned out to be a perfect biomarker for TB in the European data set, whereas miR-196b-5p and miR-589-5p performed slightly better in the Gambian cohort (Table 4). One possible explanation for this observation is that the southeastern Brazilian gene pool (from which most of our study participants were drawn) is highly enriched in elements of both European and African origin (50). Differential expression of the three miRNA components of the classifier during TB has also been documented in previous publications using different models of M. tuberculosis infection and patient samples (Table 5) (51–55). Even though expression of let-7a-5p in CD4^+^ T cells (52) (Table 5) and M1 macrophages (Fig. 9) was regulated in the opposite direction from the downregulation observed by us in whole blood, those results do suggest that all three miRNA are strongly associated with host responses to M. tuberculosis infection.

**TABLE 5:** Summary of previous studies in which the 4 components of the proposed sncRNA classifier for active TB were observed to be differentially expressed during M. tuberculosis infection^*a*^

Component of the 4-sncRNA classifier for TB	Specimen	Type of clinical specimen/cell examined	Test groups	Direction of regulation	Reference
let-7a-5p	Human U937 macrophages	Macrophages recombinantly expressing the M. tuberculosis antigen Hsp16.3	Wild type vs Hsp16.3 recombinant	↓	Meng et al. 2014 (51)
	Peripheral blood	CD4^+^ T cells	Control vs TB	—	Fu et al. 2014 (52)
			LTBI vs TB	↑	
	Peripheral blood	PBMC	Control vs TB	↓	Fu et al. 2019 (53)

miR-196b-5p	Peripheral blood	Serum	Control vs TB	↑	Zhang et al. 2014 (54)

miR-589-5p	Peripheral blood	Serum	Control vs DS-TB	—	Wang et al. 2016 (55)
			Control vs MDR-TB	↑	
			DS-TB vs MDR-TB	↑	

SNORD104	None (no available studies)				

a Symbols: ↓ = downregulation; ↑ = upregulation; — = no DE. Abbreviations: LTBI = latent tuberculosis infection; TB = tuberculosis; DS-TB = drug-sensitive TB; MDR-TB = multidrug-resistant TB; DE = differential expression.

### Comparison with other classifiers for TB.

Our report lends further support to the use of diagnostic classifiers consisting of two or more molecules. Other studies have identified multi-miRNA classifiers for TB by using either sera (6-miRNA classifier [56] or 15-miRNA classifier [57]) or whole blood (4-miRNA classifier [15]). These classifiers had lower sensitivities (≤91%) than our classifier (100%) and specificities ranging between 78.57% (57) and 91.8% (56). It may appear surprising that there is no common miRNA signature among these classifiers (including ours). Differences in study populations, clinical status in the TB spectrum (since a binary nature is an old-fashioned concept), blood fractions assayed, preanalytic protocols, transcriptomic assay (microarray or RNAseq), sequencing depth, and statistical methods and stringency may all affect the composition of the identified signatures. Clearly, this variance strongly suggests the need for unified study protocols and analytic workflows in future studies.

### Limitations and strengths.

This exploratory study was clearly limited by the small group sizes (in particular, by the sizes of the treated LTBI and TB groups), although it did not differ much from previous similar studies in this regard (15, 43, 55). Clearly, additional validation is required before advancing specific sncRNA biomarkers along the pathway to clinical application. The major strengths of this study are the fact that it was conducted in a real-life TB transmission setting, the availability of detailed clinical data, the use of both TST and IGRA to define as LTBI only the doubly positive cases, and the inclusion of subjects with LTBI who had completed prophylactic treatment. In addition, we were able to validate aspects of the 4-sncRNA classifier by comparison with published data sets, a cellular infection model, and expression of two previously validated mRNA biomarkers.

In summary, this first report on expression changes of all four major classes of sncRNA in M. tuberculosis infection suggests that, in addition to miRNA, both piRNA and snoRNA also play important roles in the host response to M. tuberculosis infection and that multi-sncRNA classifiers may prove useful for the identification of specific subgroups of humans with M. tuberculosis infection.

## MATERIALS AND METHODS

### Study design.

Participants were recruited between March 2010 and August 2013 in the context of a close-contact study conducted in the TB Control Program of Clementino Fraga Filho University Hospital (CFFUH) (58). A subset of the blood samples had been analyzed previously for mRNA expression (30), and *NPC2* was identified as a biomarker for TB. The study protocol was approved by the Ethics Committee of the Oswaldo Cruz Foundation and CFFUH under registration codes 560-10 and 183-10, respectively. Verbal and written informed consent was obtained before inclusion of patients in the study.

### Study site.

The CFFUH is a tertiary health care unit and a reference center for TB located in Rio de Janeiro (RJ), which has the second highest TB incidence (63.5/100,000 inhabitants) among the Brazilian states, representing a rate twice as high as the national average (33.5/100,000 inhabitants) (59).

### Participants and diagnostic groups.

According to the Brazilian Ministry of Health (BMH) guidelines, the screen to detect LTBI among recent contacts comprises a clinical evaluation by a physician specializing in pulmonary diseases, a chest X-ray (CXR), and a tuberculin skin test (TST). Additionally, as part of the close-contact study (58), blood was collected for short-term (st) and long-term (lt) IGRA. st-IGRA was performed by stimulating whole blood with the M. tuberculosis antigen ESAT6:CFP10 (expressed as a fusion protein) for 22 h, whereas lt-IGRA involved stimulating peripheral blood mononuclear cells (PBMC) with this antigen for 5 days. Subjects reporting cough, hemoptysis, fever, weight loss, dyspnea, or chest pain were classified as showing symptoms consistent with TB. The pulmonologist evaluated the CXR using the American Thoracic Society criteria for the radiographic diagnosis of TB (29) and recorded the presence/absence of findings suggestive of TB; radiographic alterations with no clear diagnosis were classified as representing unspecific abnormalities. LTBI subjects were offered 6 months of isoniazid (INH) preventive treatment (IPT). Active TB cases were treated with first-line anti-TB agents. All treatments and clinical evaluations were provided free of charge and on a voluntary basis and were carried out according to BMH guidelines. Cases were defined as follows. ExC had had recent close contact with a TB index case patient and had negative TST and IGRA results, indicating the absence of M. tuberculosis infection. rCt with negative TST results but positive IGRA results (or vice versa) were classified as indeterminate and were not included in the present RNA analysis. LTBI was defined as (i) the presence of a TST induration with a diameter of ≥5 mm measured 72 h after intradermal injection of M. tuberculosis purified protein derivative (PPD) and (ii) a positive IGRA response (to st-IGRA or lt-IGRA or both). If indicators of active disease were observed on CXR, (iii) the absence of acid-fast bacilli (AFB) and negative results of Lowenstein-Jensen (LJ) culture of clinical specimens were also required. Both groups were followed for 1 year, resulting in the identification of two incident TB cases. LTBItt consisted of LTBI patients (TST positive [TST^+^]/IGRA^+^ at enrollment) who had completed a 6-month course of IPT. Their blood samples were collected ≥2 months after the end of IPT (TST/IGRA were not repeated). Active TB was defined as (i) respiratory symptoms suggestive of TB and/or (ii) detection of AFB and/or positive LJ culture in sputum, bronchoalveolar lavage fluid, or a biopsy specimen followed by (iii) remission of symptoms upon anti-TB chemotherapy. Their blood samples were obtained before initiation of treatment. Whole blood was collected in PAXgene RNA tubes (PreAnalytiX; SWZ) and stored at −80°C for <2 years before RNA extraction.

### sncRNA libraries.

Small (≥18-nt) RNA extraction and purification were performed as described previously (30). A 1-μg volume of RNA was used for cDNA library preparation (TruSeq small RNA sample preparation kit; Illumina, San Diego, CA) following the manufacturer’s protocols. RNAseq was performed on an Illumina HiSeq 2500 sequencing system (Illumina, San Diego, CA), generating 50-bp single reads and ∼16 million reads passing the filter for each sample.

### Preprocessing and differential expression.

The FASTQ files were preprocessed (FastQC 0.11.2), and adaptors were trimmed (Cutadapt 1.7.1) (60), aligned to the human genome (STAR 2.4.1d) (61), counted (featureCounts 1.4.6) (62), normalized, and evaluated for differentially expressed (DE) transcripts using DESeq2 (v. 1.16) (63) on the Oasis 2.0 Web platform (64). The DESeq2 normalization metric is based on the median-of-ratios method, which accounts for sequencing depth and RNA composition (63). Normalized counts were used as input for all analyses. The DE analysis uses shrinkage estimators to infer dispersions and fold change (FC) data to facilitate interpretation of results (63). The Wald tests were used to infer *P* values for the multiple-group or pairwise comparisons after negative binomial generalized linear model (GLM) fitting, which included adjustments for the potentially confounding covariates age, sex, and RNAseq batch. This was followed by application of the Benjamini-Hochberg (BH) adjustment to estimate the false-discovery rate (FDR) among the DE sncRNA (BH-adjusted *P* values [*P*-adj]).

### Gene set enrichment analysis (GSEA) adapted for miRNA.

Enrichment analysis was performed for pathways and immune-related cell types, using the miRNA Enrichment Analysis and Annotation Web tool (65).

### Reanalysis of public miRNA microarray data sets.

Raw (data set GSE39163) or background-corrected (GSE34608) files were downloaded from the Gene Expression Omnibus (GEO) using the getGEO function from the *GEOquery* (v. 2.48.0) (66) R (v. 3.5.1) package (67). Next, expression intensities were normalized and background corrected (only for data set GSE39163) with the Robust MultiArray Average (RMA) method (68, 69) implemented in the AgiMicroRna (70) R package. Control probes were then filtered out, and miRNAs were named according to miRBase v.21 using the miRNAmeConverter (71) package. Duplicated identifiers (IDs) were removed by keeping the miRNA with the greatest mean expression level across all samples.

### *NPC2* and *BATF2* mRNA expression.

Data for these two mRNAs were extracted from the long RNA data set, which had been obtained by next-generation sequencing (NGS) from the same total RNA as the sncRNA data set (30). DESeq2 (v. 1.16) was used to obtain the normalized expression values, including adjustments for age, sex, and RNAseq batch (unpublished results).

### Primary cell isolation and *in vitro* differentiation.

Buffy coats from blood of healthy donors (HD) were provided by a blood bank (Blutbank Springe, Germany), and primary human PBMC were isolated using Ficoll (Biocoll, Merck) density gradient centrifugation. CD14^+^ monocytes were isolated by magnetically activated cell sorting (MACS) and differentiated for 5 days in serum-free medium to M1-like macrophages (M1) by adding 80 U/ml of GM-CSF (granulocyte-macrophage colony-stimulating factor; Miltenyi) or to M2-like macrophages (M2) by adding 100 ng/ml M-CSF (macrophage colony-stimulating factor; Miltenyi). A total of 1 × 10^6^ cells were plated in triplicate in 24-well tissue culture plates (Falcon) and cultured in 1 ml of CellGro medium (CellGenix) at 37°C and 5% CO_2_.

### *In vitro* infections.

M. tuberculosis strain H37Rv was grown to mid-log phase in 5 ml of Middlebrook 7H9 (Difco) liquid culture medium supplemented with 0.5% glycerol, 0.15% Tween 80, and 10% oleic acid-albumin-dextrose-catalase (BD Biosciences). Bacteria were then washed twice with 45 ml phosphate-buffered saline (PBS; Gibco). Bacterial density was measured with a spectrophotometer at an absorbance of 600 nm.

### Targeted quantification of let-7a-5p, miR-196b-5p, miR-589-5p, and SNORD104 expression.

Total RNA was extracted from cells by the use of Qiazol reagent (Qiagen) and an miRNeasy RNA extraction kit (Qiagen), using on-column RNase-free DNase (Qiagen) treatment. RNA yield was measured with a NanoDrop S1000 spectrophotometer (Thermo Scientific), and 50 ng was then reverse transcribed using a miRCURY LNA RT kit (Qiagen). Quantitative real-time PCR (RT-qPCR) was performed using a SensiFAST SYBR kit (Bioline). The primers used to amplify let-7a-5p (catalog no. YP00205727), miR-196b-5p (YP00204555), miR-589-5p (YP00205675), SNORD104 (PPH82094A), and U6 snRNA (U6; YP00203907) were obtained from Qiagen. RT-qPCR was carried out in a LightCycler 480 instrument (Roche). Relative expression levels were calculated using the 2^(−ΔΔ^*^CT^*^)^ method (72) with U6 as an internal control. We included the following controls in each PCR run: a synthetic RNA spike-in (to evaluate the efficiency of reverse transcription and RT-qPCR), a non-cDNA control (to detect primer-dimer formation), and non-RT RNA (to check for genomic DNA contamination).

### Data analysis.

The R environment (67), Prism5 software (GraphPad Software, La Jolla, CA), and the Web-based tools MetaboAnalyst (73) and Clusvis (74) were used for statistical analyses and graphics. For principal-component analysis (PCA) and generation of heat maps with individual profiles, the expression values were log transformed, median normalized, and scaled according to the Pareto scaling method (75). The R package “cluster” (v. 2.0.7-1) (76) was used for hierarchical clustering analysis based on a scaled Euclidean distance measure (Ward linkage) (77). Heat maps with group expression profiles were plotted using the average values of the normalized expression data. Univariate and multivariate ROC curve analyses were performed using R packages easyROC (78) and MetaboAnalyst (73), respectively. “Potential biomarkers” were defined as those sncRNA having an area under the ROC curve (AUROC) with a lower-bound 95% confidence interval (CI) value of ≥0.50 and an asymptotic significance *P* value of <0.05. SncRNA with AUROC values of >0.9 were defined as “highly accurate biomarkers.” A support vector machine (SVM) algorithm was applied with Monte-Carlo cross-validation to select optimal combinations of biomarkers. Then, the performance of the resulting combinations was cross-validated by the use of a logistic regression model and a predicted class probability analysis in which values between 0 and 1 represent the chances of being allocated in the disease group for 50 iterations. Decision tree analysis was performed with the R package *rpart* (v. 4.1.15) (79) and the results visualized using the Web tool draw.io (https://www.draw.io/). We used the Mann-Whitney test or Kruskal-Wallis test (with Dunn’s test) to assess differences in continuous variables between 2 groups or between >2 groups, respectively. The T-test (one-tailed) was used to assess differential expression of *NPC2* (30) and *BATF2* (31). We performed a chi-square test (two-tailed) to evaluate sex distributions across groups. For the *in vitro* assays, the Wilcoxon matched test and Friedman test were used for the paired-group and multiple-group comparisons, respectively. Spearman Rho correlations (two-tailed) were computed to assess correlations between the quantitative TST/IGRA values and the sncRNA expression values. *P* values and *P*-adj values of <0.05 were considered statistically significant.

### Data availability.

All data are publicly available through GEO (https://www.ncbi.nlm.nih.gov/geo/) under accession number GSE131174.
