# Supplementary figures and images for: Reprogramming of Small Noncoding RNA Populations in Peripheral Blood Reveals Host Biomarkers for Latent and Active Mycobacterium tuberculosis Infection
Source: mBio. 2019 Dec 3;10(6):e01037-19. doi: 10.1128/mBio.01037-19 (PMC6890987; doi:10.1128/mBio.01037-19)

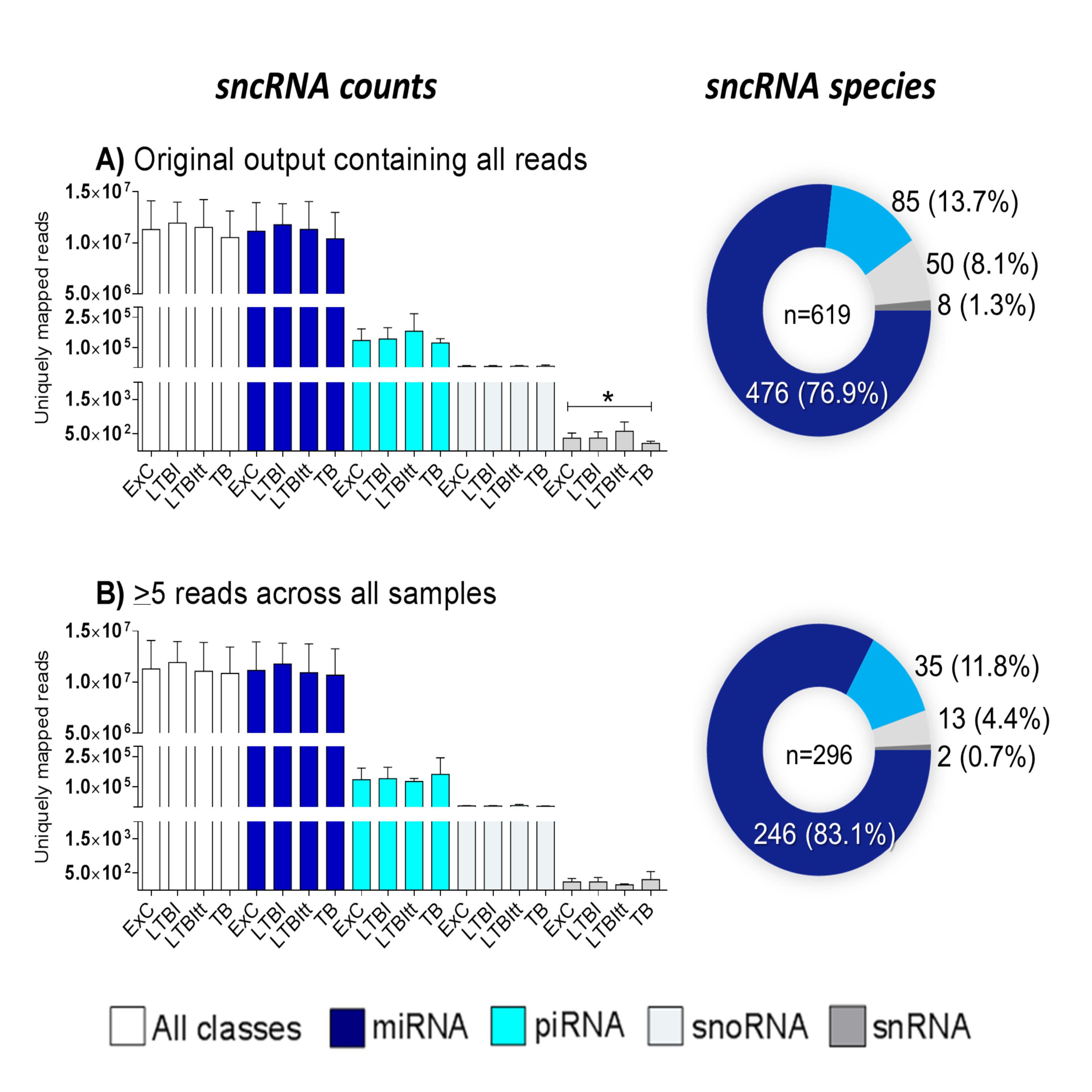

Supplement: FIG S1 [file mBio.01037-19-sf001.tif]

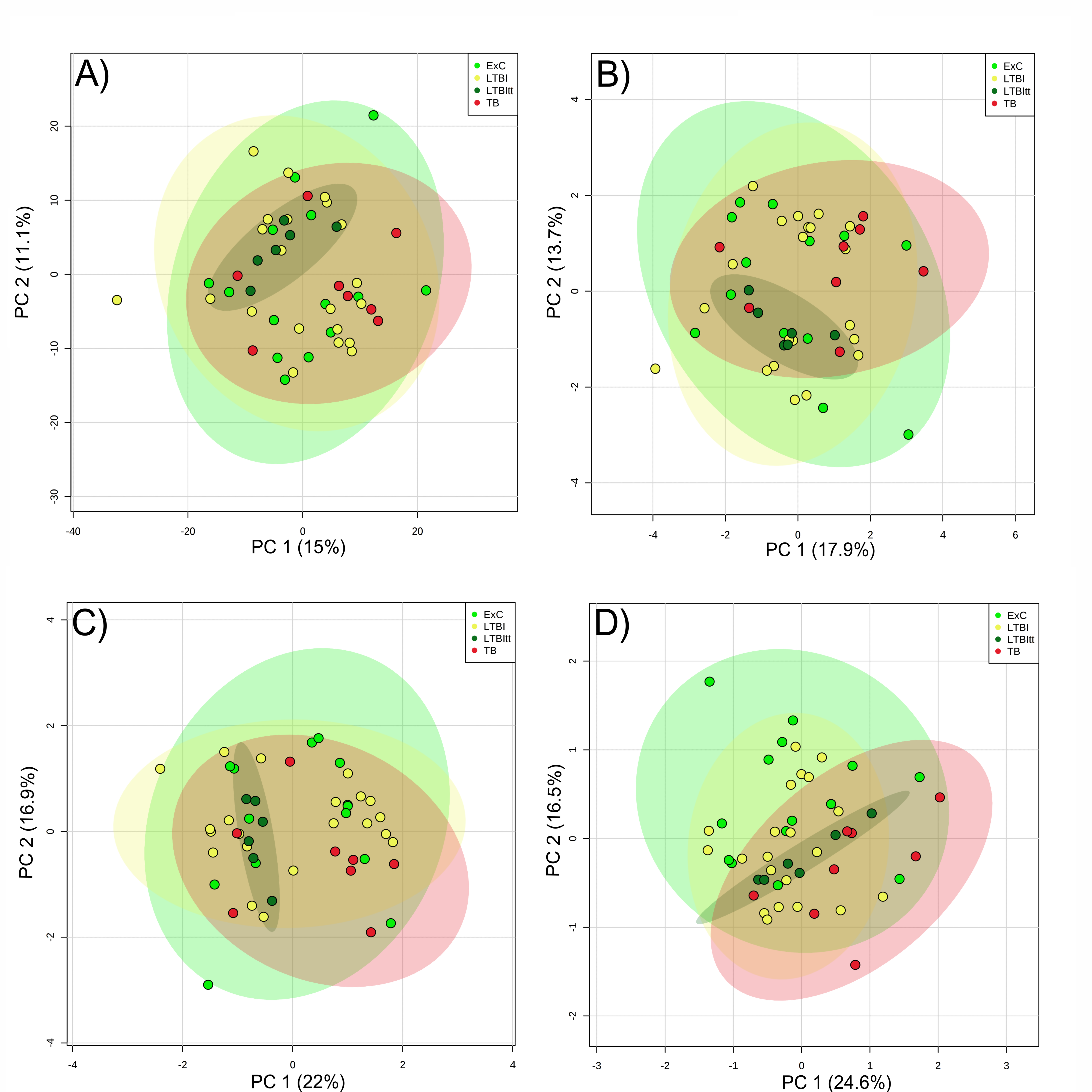

Supplement: FIG S2 [file mBio.01037-19-sf002.tif]

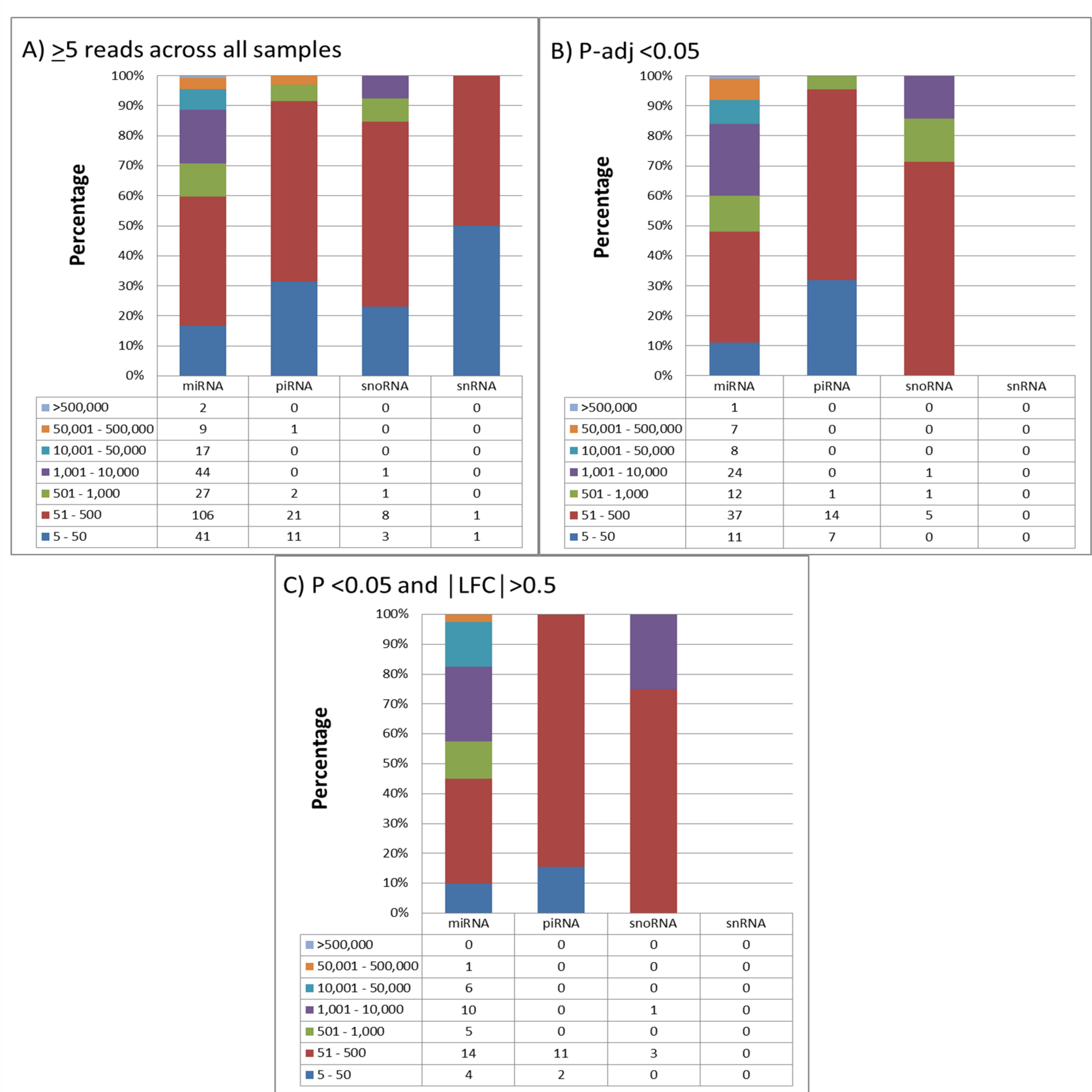

Supplement: FIG S3 [file mBio.01037-19-sf003.tif]

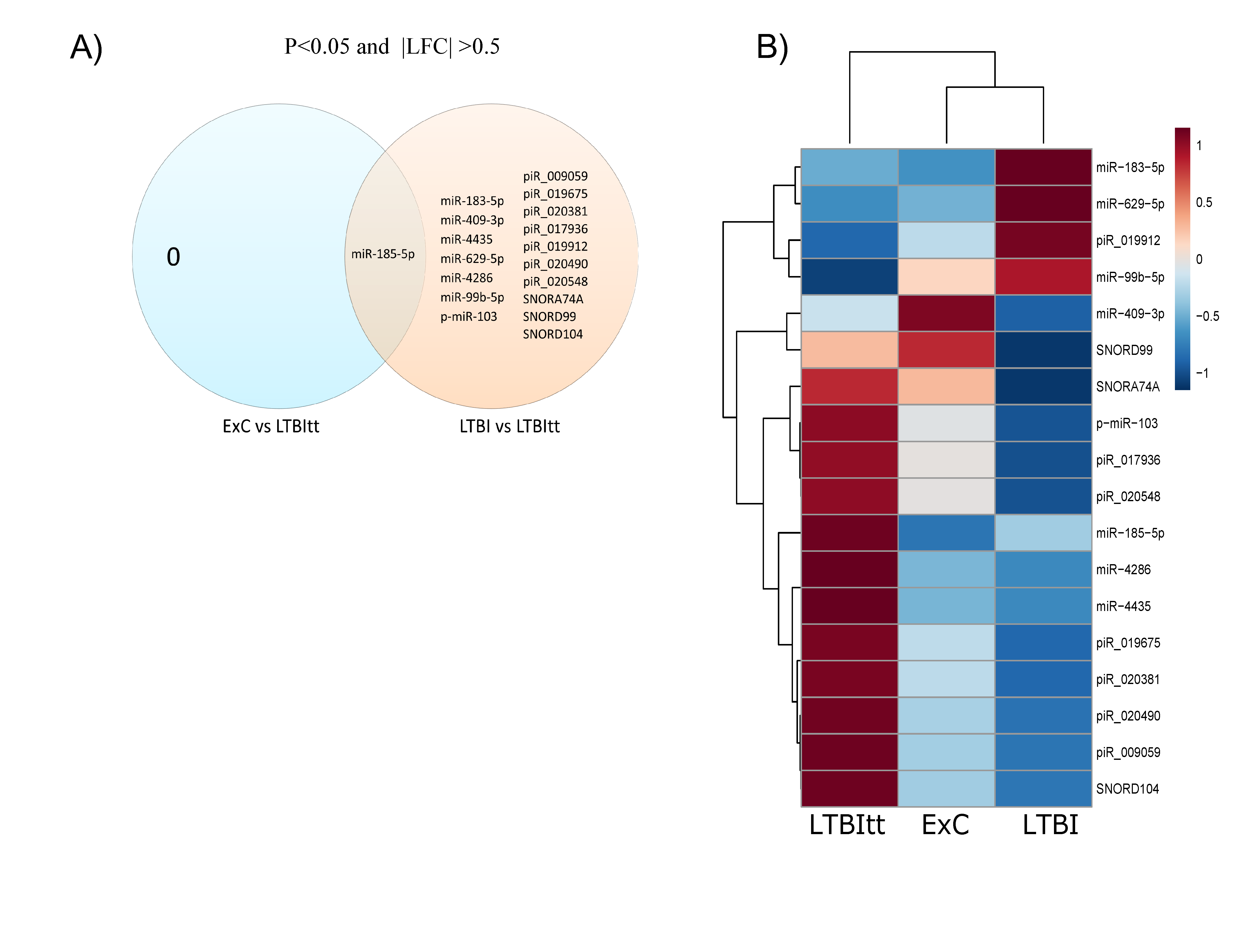

Supplement: FIG S5 [file mBio.01037-19-sf005.tif]

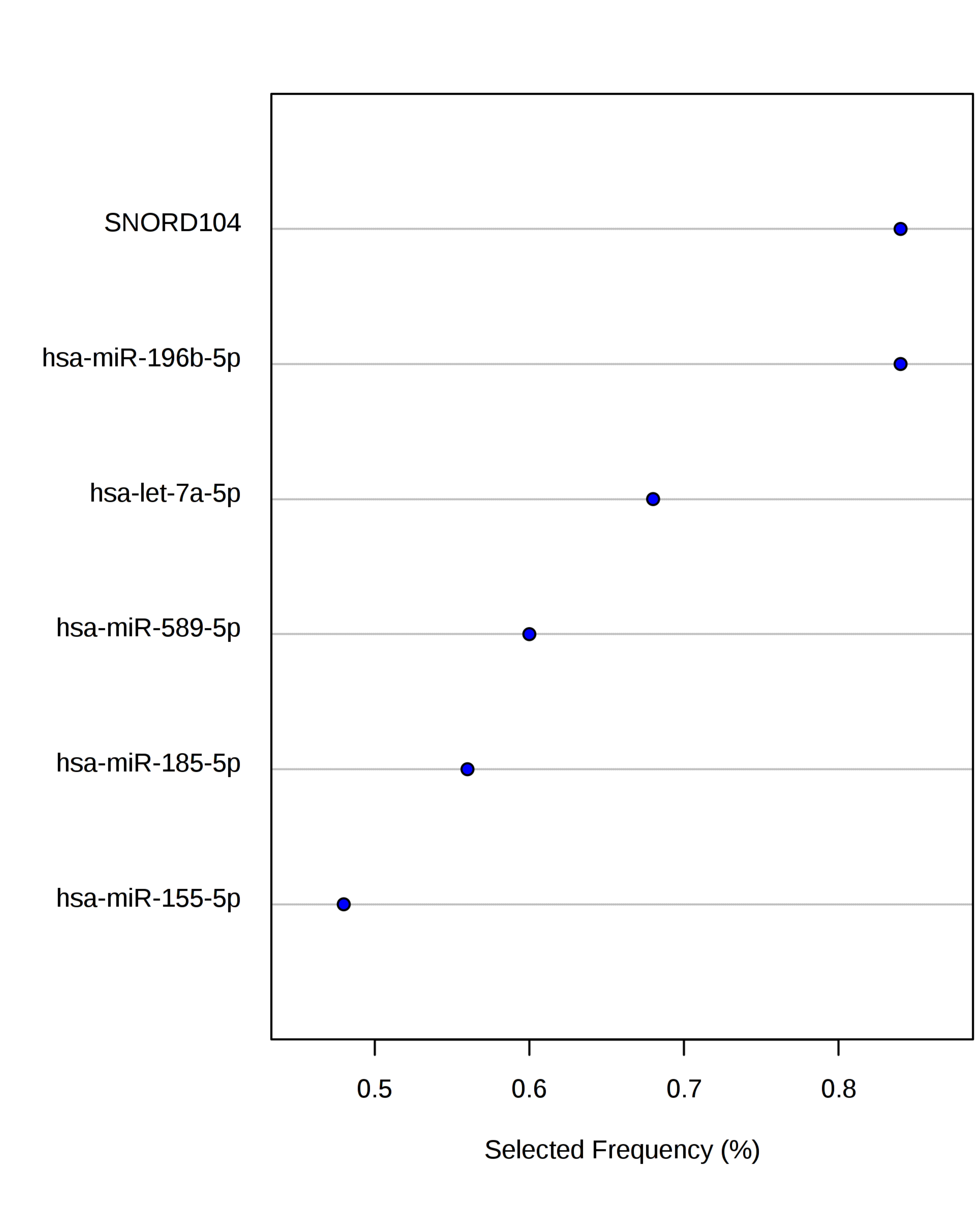

Supplement: FIG S4 [file mBio.01037-19-sf004.tif]
